# Structural and Functional Studies on Key Epigenetic Regulators in Asthma

**DOI:** 10.3390/biom15091255

**Published:** 2025-08-29

**Authors:** Muhammad Fakhar, Mehreen Gul, Wenjin Li

**Affiliations:** 1 College of Civil and Transportation Engineering, Shenzhen University, Shenzhen 518060, China; mfakhar@bs.qau.edu.pk (M.F.); mehreengull@bs.qau.edu.pk (M.G.); 2 Institute for Advanced Study, Shenzhen University, Shenzhen 518060, China

**Keywords:** asthma, epigenetic regulation, p300/CBP, SIRT family, DNMT1, DNMT3A, YTHDF1/2, IGF2BP2, precision medicine

## Abstract

Asthma is a chronic inflammatory airway disease influenced by both genetic and environmental factors. Recent insights have underscored the pivotal role of epigenetic regulation in the pathogenesis and heterogeneity of asthma. This review focuses on key epigenetically important regulators categorized as writers, erasers, and readers that govern DNA methylation, histone modifications, and RNA modifications. These proteins modulate gene expression without altering the underlying DNA sequence, thereby influencing immune responses, airway remodeling, and disease severity. We highlight the structural and functional dynamics of histone acetyltransferases (e.g., p300/CBP), histone deacetylases (e.g., SIRT family), DNA methyltransferases (DNMT1, DNMT3A), demethylases (TET1), and methyl-CpG-binding proteins (MBD2) in shaping chromatin accessibility and transcriptional activity. Additionally, the m6A RNA modification machinery including METTL3, METTL14, FTO, YTHDF1/2, IGF2BP2, and WTAP is explored for its emerging significance in regulating post-transcriptional gene expression during asthma progression. Structural characterizations of these proteins reveal conserved catalytic domains and interaction motifs, mirroring their respective families such as SIRTs, p300/CBP, DNMT1/3A, and YTHDF1/2 critical to their epigenetic functions, offering mechanistic insight into their roles in airway inflammation and immune modulation. By elucidating these pathways, this review provides a framework for the development of epigenetic biomarkers and targeted therapies. Future directions emphasize phenotype-specific epigenomic profiling and structure-guided drug design to enable precision medicine approaches in asthma management.

## 1. Introduction

### 1.1. Background on Asthma

Asthma, a chronic inflammatory respiratory condition, can manifest as symptoms like wheezing, coughing, airflow limitation and chest tightness instigated through chronic airway inflammation, tissue remodeling, mucus hypersecretion and bronchial hyperresponsiveness [1,2,3]. Based on the Global Burden of Disease Study 2019, the asthma affected 10.9% of the population in the United States, encompassing approximately 34 million individuals. This rate significantly surpasses that recorded in China. Despite this, asthma in China often goes undiagnosed and undertreated. The China Pulmonary Health study, a national cross-sectional analysis, indicated that around 45.7 million adults in China were impacted by asthma, yielding a prevalence of 4.2% in 2015 [4]. Over the past two decades, there has been a significant rise in both the incidence and prevalence of asthma worldwide [5]. Asthma impacts more than 300 million people globally and is associated with substantial health risks [6]. Although numerous advancements have been made in treating and diagnosing asthma, a large population still suffers from this chronic, non-communicable nature. It remains a major global health concern, and approximately 10% of asthma patients do not respond adequately to standard therapy [7,8]. These statistics point to asthma becoming one of the most significant health challenges of our time, emphasizing the urgent need for effective prevention, therapeutics, and disease management. Research suggests that the development of asthma and allergic conditions is multifactorial, with both genetic and environmental factors playing key roles. Environmental factors affect the epigenome, so epigenomic and transcriptomic profiling may be useful in addition to genomic profiling for asthma prediction [9].

### 1.2. Role of Epigenetics in Asthma

Compared to genetics, epigenetics affects gene expression without altering DNA sequence [10]. Epigenetic changes may be triggered by environmental factors, including prenatal smoking and postnatal pollutants, nutrients, and drugs [11]. Asthma is considered a heritable disease according to numerous genetic studies; however, the rising incidence of asthma particularly in recent years cannot be solely attributed to genetic elements [12]. Epigenetics is regarded as a key contributor to asthma pathogenesis [13]. Numerous studies have highlighted the importance of epigenetic modifications such as DNA methylation, histone modifications, and RNA modifications in regulating gene expression patterns relevant to asthma [14]. Yang et al. (2017) demonstrated substantial differences in DNA methylation patterns between asthmatic and non-asthmatic individuals, particularly in genes associated with immune regulation and airway inflammation [15]. This finding underscores the potential role of epigenetic dysregulation in asthma susceptibility and severity. Epigenetic modifications take place in prenatal development, early childhood, and adolescence, as these life stages represent vulnerable periods to various asthma inductions [16]. In addition to the skin, respiratory tract is directly exposed to outer atmosphere, initiating epigenetic changes to nasal, buccal mucosa and bronchial epithelium [16]. DNA methylation plays a critical role in asthma by modulating gene expression in a cell-type-specific manner. Studies reviewed by Thibeault and Laprise (2019) [17] emphasize that whole blood methylation patterns largely reflect eosinophil activity, which may mask signals from other immune or respiratory cells. By analyzing isolated cell types such as eosinophils, monocytes, B cells, airway epithelial cells, and smooth muscle cells researchers identified distinct methylation signatures linked to asthma pathophysiology. These studies primarily used the Illumina 450K array in epigenome-wide association studies (EWAS). The findings underscore that asthma-associated DNA methylation is highly dependent on the specific cell type analyzed, requiring cell purification over bulk tissue analysis [17].

### 1.3. Significance of Studying Epigenetic Regulators

Epigenetic modifications, which include DNA methylation, histone modifications, chromatin remodeling, and RNA regulation are dynamically regulated by a myriad of epigenetically important proteins, which act as writers, erasers, and readers of epigenetic marks [18]. Dysregulation of these proteins has been implicated in various diseases, highlighting the importance of studying their structures, functions, and molecular mechanisms. This review highlights how investigating the structure and function of key epigenetic regulators can enhance our understanding of asthma pathogenesis, identify novel therapeutic targets, and support precision medicine.

### 1.4. Overview of Epigenetic Mechanisms

**Figure 1:**
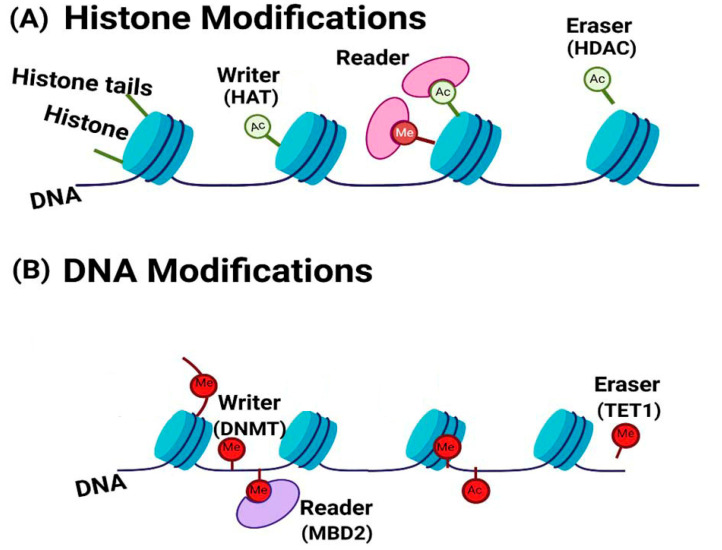

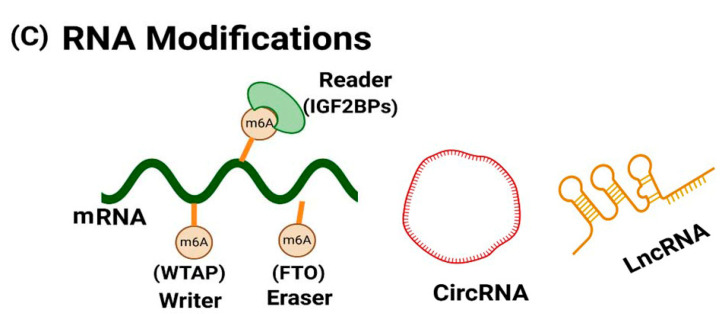
A basic summary of the primary epigenetic modifications. (**A**) Chromosomal DNA wraps around histone proteins, forming nucleosomes. The accessibility of nucleosomes to nuclear factors is partially controlled by modifications to the tails of histone proteins. These modifications primarily include acetylation and methylation. Acetylation reactions, facilitated by writer enzyme histone acetyltransferase (HAT). This process neutralizes the positive charges on histone, resulting in a less condensed chromatin structure that is conducive to transcription. Conversely, deacetylation reactions, mediated by histone deacetylase (HDAC), remove acetyl groups from histone tails. This increases chromatin packaging, leading to a tighter conformation that inhibits DNA transcription. (**B**) In DNA modification methylation involves the addition of a methyl group to the carbon-5 position of cytosine within regions known as CG Islands, facilitated by DNA-methyltransferase (DNMT) enzymes. Methylation of CpG islands can lead to gene silencing by either blocking the binding of transcription factors (TFs) to the promoter or by promoting the binding of proteins, such as MeCpG-binding proteins (e.g., MBDs), that specifically recognize methylated CpGs. Furthermore, DNA methylation can reinforce certain histone modifications, such as demethylation and deacetylation, thereby establishing a mechanism for perpetuating these epigenetic marks. (**C**) RNA modification including mRNA and non-coding RNAs (ncRNAs). Non-coding RNAs are typically categorized as small or long ncRNAs. Functionally, they fall into two main groups: constitutively expressed housekeeping molecules and regulatory molecules, including microRNAs (miRNAs) and long non-coding RNAs (lncRNAs). The modification of m^6^A in mRNA is regulated by ‘writers’ ‘readers’, and ‘erasers’. Writers such as METTL3, METTL14, and WTAP regulate m^6^A methylation. RNA m^6^A demethylation is prompted by eraser proteins such as FTO while reader proteins like IGF2BPs recognize these modifications and regulate downstream RNA processing and stability. This figure was generated in this study.

Epigenetics refers to heritable, reversible changes in gene expression that do not involve alterations to the DNA sequence [19]. Within the human genome, approximately 80% of DNA is organized into nucleosomes, with the remaining forming linker regions between these nucleosomes. These nucleosomes are subsequently organized into chromosomes, forming dense three-dimensional structures [20]. Histone proteins constitute the core components of nucleosomes, and they endure numerous posttranslational modifications (PTMs), including phosphorylation, sumoylation, ubiquitination, methylation and acetylation. These PTMs, particularly present in critical regulatory genomic regions such as promoters or enhancers, can modulate DNA accessibility to the transcriptional machinery, thereby influencing the gene’s transcriptional status as active, poised, or silenced. For instance, histone acetylation, which is facilitated by histone acetyltransferases (HATs), typically promotes gene transcription by loosening chromatin structure, whereas histone deacetylases (HDACs) remove these acetyl groups, leading to chromatin condensation and transcriptional repression [14,21]. DNA methylation encompasses the enzymatic addition of a methyl group to the cytosine ring of DNA, representing another form of epigenetic modification. DNA methylation, typically associated with gene repression, is catalyzed by DNA methyltransferases, while demethylation is performed by TET enzymes [22]. In addition to traditional epigenetic modifications, post-transcriptional modifications have also been observed in both coding and non-coding RNA molecules, including rRNA, tRNA, mRNA, miRNA, circular RNA (circRNA), and various long non-coding RNAs (lncRNAs), all of which contribute to the epigenetic modulation of gene expression [23]. For instance, miRNAs operate through attaching mature mRNA molecules in the cytoplasm, resulting in mRNA degradation or decreased translational efficiency by ribosomes [24,25]. The overall summary of epigenetic modifications is illustrated in Figure 1. Epigenetic regulation plays a critical role in asthma pathogenesis, influencing gene expression through DNA methylation, histone modifications, and RNA modification [26,27]. 

## 2. Role of Key Epigenetic Regulators in Asthma Pathogenesis

Several key proteins play crucial roles in epigenetic regulation, including DNA modification, histone modification, and RNA modification. In the context of airway inflammation and asthma, gene expression is regulated by a dynamic writer–eraser–reader system. Specifically, in RNA modification, writers like METTL3 deposit m^6^A, erasers such as FTO remove these marks, and reader proteins like IGF2BPs and YTHDFs interpret them to regulate RNA stability and the expression of inflammatory mediators. RNA modifications, notably m^6^A, play a pivotal role in governing immune responses, as alterations in RNA modification regulators within immune cells can influence the expression of genes involved in immunity, inflammation, and pathogen defense. This mechanism contributes to the post-transcriptional control of gene expression in asthma pathogenesis [28]. Similarly, a wide array of regulatory proteins act as writers, erasers, and readers in DNA and histone modifications, and their functions are summarized in Table 1.

## 3. Epigenetic Regulators in Asthma: Writers, Readers, and Erasers of Histone Modifications

Histone modifications, including acetylation, methylation, phosphorylation, ubiquitination, and sumoylation, are key regulators of chromatin structure [51]. Histone acetylation takes place at lysine residues positioned on the N-terminal tail of histones, regulated by histone acetyltransferases (HATs) and histone deacetylases (HDACs), which have opposing effects [52,53]. Human HATs can be sorted into 6 main orders based on the structure, features and mode of action of their catalytic domains. Lysine acetyltransferases 2A and 2B are members of the GCN5-related N-acetyltransferase family, which is a class of HATs that acetylate transcription factors and histones. They control cell cycles, centrosome’s activity, DNA replication and repair. The MYST family has the following members KAT6A/MYST3/MOZ, KAT6B/MYST4/MORF, KAT7/MYST2/HBO1, KAT8/MYST1/hMOF, and KAT5/Tip60. This HAT group of enzymes is involved in transcription control and DNA repair. The transcriptional factor-related HAT family has the following members KAT4/TAF1/TBP and KAT12/TIFIIIC90, whereas KAT3A and KAT3B enzymes belong to the E1A binding protein p300 (EP300) and CREB binding protein (CREBBP, CBP) family [54,55,56]. The E1A binding protein p300 (p300) and p300/CBP-associated factor (PCAF) are both bromodomain-containing histone acetyltransferases (HATs) that play key roles in regulating histone modifications in airway smooth muscle cells (ASMCs), particularly in allergic inflammation of the respiratory tract. In asthma, both p300 and PCAF are associated with increased acetylation of histone H3, specifically at H3K18ac, at the CXCL8 gene promoter. This enhanced acetylation promotes CXCL8 gene expression, which is critical for the inflammatory response in asthma [57]. Lastly, KAT1/HAT1 and HAT4/NAA60 are histone acetyltransferases (HATs) located in the cytoplasmic, whereas KAT13A/SRC1, KAT13B/SCR3/AIB1/ACTR, KAT13C/p600, and KAT13D/CLOCK comprise the steroid receptor coactivators family [52,54,55,56]. Histone deacetylases are classified into 4 categories, that is Class I-IV: class I “(HDAC1, HDAC2, HDAC3, and HDAC8)”, class II “(HDAC4, HDAC5, HDAC6, HDAC7, HDAC9, and HDAC10)”, class III “(SIRT1-SIRT7)”, and class IV “(HDAC11)” [58]. HDACs of Class I, II, and IV rely on Zn^2+^ as a cofactor for their enzymatic activity, whereas the enzymatic function of class III HDACs, also known as Sirtuins, is dependent on NAD^+^ [59]. Histone acetylation “readers” are proteins that recognize acetylation marks, dynamically regulated by HATs (“writers”) and HDACs (“erasers”), and influence transcriptional processes. Dysregulation of these readers is often linked to various human diseases [60,61,62]. Ogasawara et al. in 2017 [63] highlighted the role of histone acetylation in regulating T cell function in asthma. Bcl6 suppresses IL-4 expression in murine memory Th2 cells by binding to intron 2 of the Il4 locus, an effect modulated by IL-33 and associated with changes in H3K9 and H3K14 acetylation [63]. Table 1 summarizes key histone acetyltransferases (HATs) such as p300/CBP and KAT2A which act as writers and histone deacetylases (HDACs) from class I and III, which function as erasers of histone modifications involved in asthma pathogenesis. These enzymes regulate chromatin accessibility and the expression of inflammatory genes central to asthma. Altered expressions or activity of these specific HATs and HDACs are linked to airway inflammation, immune cell activation, and disease severity. Some of these enzymes are detailed in terms of their structure and function, emphasizing their roles in modulating chromatin and regulating asthma-related gene expression.

### 3.1. Functional Roles of CREB-Binding Protein and E1A-Associated Protein p300 in Asthma

Barnes et al. has investigated that the levels of histone acetyltransferases (HATs), specifically CREB-binding protein (CBP) and E1A-associated protein p300 (p300) have been raised, while the levels of histone deacetylases (HDACs) were notably reduced in the bronchial biopsy samples from asthmatic patients in comparison to those from healthy persons. These changes favor the release of inflammatory factors, which are crucial for the amplification and persistence of inflammatory responses. This mechanism triggers chronic inflammation and the remodeling of the airway [64,65]. Research has shown that ORMDL3′s promoter region has p300 HAT binding sites. In order to maintain the ORMDL3 gene’s basic transcriptional activity, p300 helps transcription factors like ETS1 and STAT6 via binding to the promotor region of ORMDL3 [66,67]. Increased expression of ORMDL3 has been observed in over one-third of children under the age of 7 with asthma [68]. Previous studies have shown that ORMDL3 contributes to airway inflammation and remodeling and also promotes bronchial epithelial–mesenchymal transition (EMT) [69]. An in vitro study using asthmatic mice revealed that histone hyperacetylation at the ORMDL3 gene is linked to asthma, and the resulting increase in ORMDL3 expression is associated with airway remodeling [68]. Consequently, targeting the overexpression of ORMDL3 and uncovering the mechanisms that regulate its activity may offer promising avenues for developing new asthma therapies. p300 is a classic endogenous histone acetyltransferase [70]; it is extensively conserved across species and is integral to cell cycle regulation, cellular transformation, and apoptosis. Currently, three key biological functions of p300 have been identified. (1) Acetylation of histone tails: p300 facilitates transcription via the catalytic function of its KAT domain, which acetylates nucleosomal histones at promoters, leading to chromatin remodeling and relaxation, thereby enhancing DNA accessibility to other critical regulators [71]. Due to its capacity to alter chromatin structure through histone acetylation, p300 is characterized as a “writer” of the epigenetic code [72]. (2) As p300 acetylase modifies nonhistone transcription factors via acetylation thus augmenting their activity. (3) p300 acts as a transcriptional activator by recruiting transcription factors to the promoter localities of target genes, thus enhancing transcription [68]. Consequently, it is essential to explore whether histone acetylation mediated via p30 has a key role in the overexpression of ORMDL3 in asthma. Chen et al. in 2020 found that increased levels of p300/CBP, a histone acetyltransferase, were associated with elevated inflammatory cytokines and poor prognosis in acute respiratory distress syndrome, highlighting its role in inflammation and disease outcome [73]. Given that p300/CBP also plays a key role in asthma-related inflammation and airway remodeling, these findings underscore a shared epigenetic mechanism, suggesting that insights from ARDS may help identify novel therapeutic targets for severe or steroid-resistant asthma.

### 3.2. Structure of CREB-Binding Protein and E1A-Associated Protein p300

**Figure 2:**
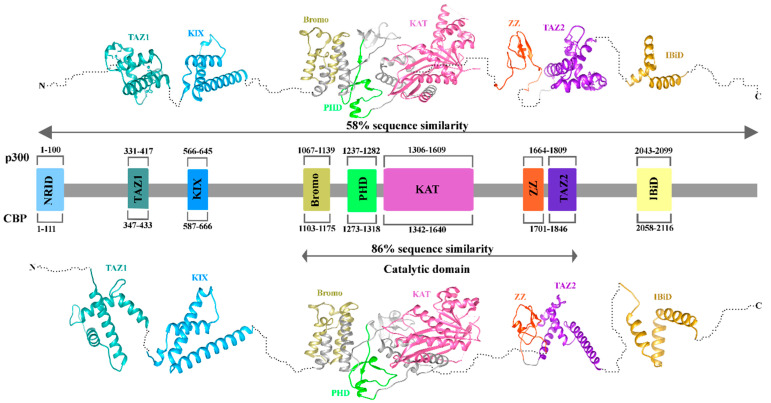
Structure of p300 and CBP. The p300 and CBPs consist of 2414 and 2442 amino acids, respectively, and both have a molecular weight of around 265 kilodaltons. The 58% sequence similarity has been observed within their respective domains of both proteins. Both proteins comprise various domains like on N terminal there is nuclear receptor interaction domain (NRID) followed by three regions rich in cysteine and histidine (C/H1 to C/H3), which feature zinc finger transcriptional adapters TAZ1 and TAZ2, and C/H2, which contains a homeodomain (PHD), a KIX domain, a bromodomain, a lysine acetyltransferase domain (KAT), and an interferon-binding transactivation domain (IBiD). The locations of the various domains are specified in relation to their positions within the amino acid sequence. The catalytic domain has exhibited significant conservation throughout evolution, with 86% similarity between p300 and CBP encompassing the KAT domain and adjacent areas. In asthma, p300/CBP interaction domains recruit ETS1 and STAT6 to the ORMDL3 promoter, while the KAT domain acetylates histones, sustaining ORMDL3 expression and promoting airway inflammation and remodeling. This figure was generated in this study.

Transcription factors (TFs) engage with coactivators such as the Mediator complex and the acetyltransferases CBP (KAT3A, CREBBP gene) and p300 (KAT3B, EP300 gene) via their activation domains; these two paralogous lysine acetyltransferases were discovered in the 1980s and 1990s [74,75,76,77,78,79,80,81,82]. EP300 and CREBBP comprise 31 exons and extend around 87 kb and 155 kb, respectively [83,84,85]. They possess a comparable structure with distinct functional domains, encompassing various protein–protein interaction motifs, and overall exhibit 58% of sequence identity (Figure 2). A nuclear receptor interaction domain (NRID) which is capable of binding to PXXP motifs is present on the N terminal region. Three regions rich in cysteine and histidine (C/H1 to C/H3) are implicated in protein–protein interactions. The zinc finger transcriptional adapters like TAZ1 and TAZ2 are present in C/H1 and C/H3 domains, whereas the C/H3 domain further includes a ZZ zinc finger domain and C/H2 have a homeodomain (PHD). The catalytic domain has exhibited significant conservation throughout evolution, with an 86% similarity between p300 and CBP. The structure comprises a KAT domain along with adjacent sections, specifically the Bromodomain, C/H2, and C/H3 regions [86,87,88,89]. Overall, the domains of p300/CBP can be functionally grouped into interaction domains (e.g., NRID, CH1, CH2, CH3, TAZ1, TAZ2, ZZ zinc finger, and PHD) and an enzymatic domain (KAT), with the bromodomain acting as a reader module adjacent to the catalytic region. The interaction domains of p300/CBP mediate binding with transcription factors such as ETS1 and STAT6 to the ORMDL3 promoter, while the KAT (HAT) domain promotes histone acetylation to maintain transcriptional activity. These coordinated actions contribute to the upregulation of ORMDL3, thereby promoting airway inflammation and remodeling characteristics of asthma [68]. The KAT domain promotes the transcription of pro-inflammatory cytokines, while the adjacent zinc-binding domain stabilizes the protein’s structure and is essential for maintaining its enzymatic activity. Targeting these domains with selective inhibitors may suppress excessive inflammatory gene expression, offering a potential therapeutic strategy for asthma.

As shown in Figure 2, the interaction domains span extensive intrinsically disordered regions (IDRs). The full-length structure of CBP (AF-Q92793-F1) was obtained from the AlphaFold database (https://alphafoldserver.com/, accessed on 18 November 2024) while the p300 structure was predicted using the AlphaFold3 (AF3) database. Both were annotated by domain. P300 is activated through autoacetylation [86], a process modulated by cellular signaling via transcription factor activation and dimerization [90]. P300/CBP is a multipurpose protein that serves as both a “writer” and a “reader” of lysine acetylation via its KAT domain and bromodomain (BD), respectively, to sustain transcriptional activation [86,91]. This dual functionality not only amplifies its regulatory impact on gene expression but also presents a valuable therapeutic opportunity. Targeting either its catalytic or recognition activity with small-molecule inhibitors could effectively disrupt the persistent activation of pro-inflammatory genes, offering a novel strategy for asthma intervention. P300/CBP is a versatile lysine acetyltransferase capable of acetylating all four of the canonical histones [92]. There is a significant sequence similarity in other non-catalytic domains. The KIX domain promotes the binding of CREB, especially at the phosphorylated Ser133 residue, to other transcription factors, leading to the formation of a bromodomain (BD) that interacts with acetylated lysines [93]. The C terminal region of p300/CBP features an interferon-binding transactivation domain (IBiD), which encompasses a glutamine-rich domain and nuclear binding coactivator domain, succeeded by a proline-rich PxP motif [94,95].

### 3.3. Functional Roles of Sirtuins in Asthma

Sirtuins are NAD-dependent class III histone deacetylases (HDAC), consisting of seven types (Sirt1-7) in human [96], and can be categorized into four primary groups according to their sequence homology [97]. SIRT1–SIRT3 belong to class I, SIRT4 to class II, SIRT5 to class III and SIRT6 and SIRT7 to class IV [97]. A similar catalytic domain is present between all SIRTs and use NAD+ as a co-substrate. However, they differ in substrate affinities and sub-cellular localization [98,99]. SIRT1, 6 and 7 are principally found into the cellular nucleus, whereas SIRT3, 4 and 5 are mainly mitochondrial enzymes and SIRT2 is exceptional in the group, being a primarily cytoplasmic protein [100]. Sirtuins (SIRTs) play a key role in regulating inflammation via various mechanisms, such as NF-kB and NOD-like receptor thermal protein domain associated protein 3 (NLRP3) pathways [101]. Nuclear-localized SIRTs can regulate the expression of genes involved in inflammation, thereby influencing the initiation and progression of inflammatory responses [102]. Beyond their roles in aging and inflammation, SIRTs are involved in the regulation of various diseases, including cancer, cardiovascular disorders, and respiratory conditions such as asthma making them promising targets for therapeutic intervention. Given its chronic inflammatory nature, asthma is of particular concern, especially because it affects individuals across all age groups and is notably prevalent in children [103]. Current investigations have indicated that targeting SIRT1 could offer a promising new approach for asthma treatment [35,104]. It has been observed that the level of SIRT1 protein was downregulated in severe asthmatic patients [105]. SIRT1 has a pivotal role in mitigating inflammation in airway diseases such as asthma. In a study by Tang et al. (2018), SIRT1 was found to modulate inflammation by regulating IL-6 production through the Akt pathway in the context of allergic asthma [106]. Additionally, SIRT1 influences pulmonary function in asthma patients through its effect on IL-6 levels via the Akt signaling pathway [107]. Besides SIRT1, other sirtuins including SIRT2, SIRT3, SIRT6, and SIRT7 have also been linked with asthma. For example, SIRT2 aggravates allergic asthmatic inflammation, where inhibiting SIRT2 pharmacologically alleviates its severity, whereas genetic overexpression of SIRT2 worsens the allergic asthma phenotype [108]. Additionally, SIRT2 augments asthmatic related inflammation via promoting T-helper type 2 responses and macrophage polarization [36]. In contrast, raising the expression has been shown to reduce apoptosis in the bronchial epithelium and airway inflammation in asthma [37]. Allergic asthma is a persistent inflammatory disorder of the airways, marked by airway remodeling, which severely impairs airflow in the lungs [109]. SIRT6 and SIRT7′s expression levels have been elevated in human bronchial epithelial cells which were obtained from asthma patients [32]. Enhanced SIRT6 alleviates airway remodeling by modulating epithelial–mesenchymal transition in asthma [110]. Conversely, increased SIRT7 facilitates airway remodeling in asthma by modulating TGF-β1-driven proliferation and migration of airway smooth muscle cells [39]; suggesting a distinct role for SIRT6 in airway remodeling.

### 3.4. Structure of Sirtuins

The sirtuin families demonstrate structural similarity, characterized by a conserved enzymatic core that encompasses a Rossmann fold domain along with a smaller domain composed of zinc-binding and helical domains [111]. Biochemical research on sirtuin activity discloses that deacetylation takes place in a stoichiometric manner, accompanied by the hydrolysis of the bond between nicotinamide and ribose in NAD+. This process results in the creation of a temporary covalent intermediate known as an O-alkylamidate [112]. Avalos et al. (2002) proposed two distinct structure-based mechanisms for the nicotinamide cleavage reaction in sirtuins [113]. Zhao et al. in 2004 demonstrated that NAD+ binds to the sirtuin pocket in two different conformations: productive and non-productive [114]. In the productive state, NAD+ positions the positive charge of the nicotinamide ring within a conserved region called the C-site, which contains highly hydrophobic residues that alter the resonance of the delocalized electrons in the nicotinamide ring by distorting the carboxamide group. In the non-productive conformation, the nicotinamide ring does not fit into the C-site [115].

Sirt2, the primary subtype identified via Finnin et al. in 2001 [116], was the subject of the initial characterization of the Sirtuin protein structure, offering valuable insights into the sirtuin catalytic core (PDB 1J8F). Notably, the structure of Sirt2′s catalytic core serves as a nearly representative model for the core region of all sirtuin isoforms, which contributes to the high degree of conservation observed within the Sirtuin family [117]. SIRT2 comprises a total of 389 residues, with 323 amino acids successfully crystallized at a 1.7 Å resolution, providing a robust foundation for understanding its structural properties [116].

**Figure 3:**
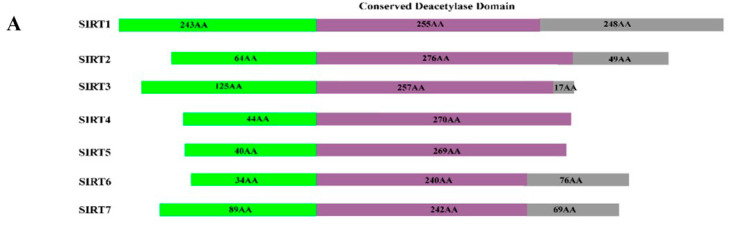

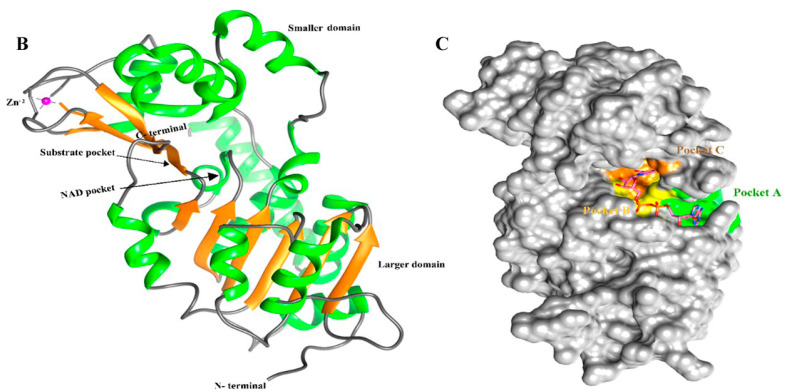
Conservation mapping of sirtuins. (**A**) The figure depicts the conservation analysis of seven sirtuin proteins (SIRT1–SIRT7), showing their conservation patterns. (**B**) Ribbon and (**C**) Surface views of SIRT2 3D structure. The conserved deacetylase domain of SIRT2, particularly its NAD+ and substrate binding pockets, plays a crucial role in modulating inflammatory pathways. The zinc tetra-thiolate motif (in gray) is crucial for maintaining the structural integrity and enzymatic activity of sirtuin. This figure was generated in this study.

As illustrated in Figure 3B, SIRT2 features an active catalytic core along with helical extension at its N-terminal. The catalytic core comprises a standard NADH-binding site, which is divided into distinct domains: a smaller one and a larger one. The larger domain encompasses an inverted Rossmann fold, a distinctive motif typical of NAD+(H) binding sites, which contains six β-strands (βI-III and βVII-IX) and six α-helices (αI, αVII, αVIII, αX, αXI, and αXII). These structural elements are aligned parallel to the β-sheet (residues “53–89”, “146–186”, and “241–356”) [118]. The smaller domain is further categorized into Zinc binding and helical subdomains. The Zn-binding domain features three antiparallel β-sheets, one α-helix, and a Zn2^+^ ion coordinated by four conserved cysteine residues (Cys195, Cys200, Cys221, and Cys224) found across all Sir2-like enzymes [116]. This zinc tetra-thiolate motif is essential for maintaining the structural integrity and enzymatic activity of sirtuin proteins. In immune cells, particularly macrophages, oxidative stress can reversibly modify these cysteine residues, impairing SIRT2′s deacetylase activity. This leads to increased acetylation of NF-κBp65 and elevated transcription of pro-inflammatory cytokines such as TNF-α, IL-1β, and IL-6, thereby amplifying inflammatory responses [119]. The helical domain comprises four helices, two short and two long. These two domains are connected through a short polypeptide chain in the smaller domain and three larger polypeptide chains in the larger domain, forming a prominent groove. NAD+ binding site is present within this groove, and the amino acids involved are highly conserved within the Sirtuin family. The NAD+ cofactor-binding pocket is divided into three regions: the adenine ribose moiety binds to the A-site, the nicotinamide ribose moiety interacts with the B- and C-sites, and the nicotinamide itself resides deep within the catalytic pocket (Figure 3C). Mutations at these sites lead to a complete loss of deacetylation activity, confirming that this large junction groove forms the core of the catalytic mechanism [116].

## 4. Epigenetic Regulators in Asthma: Writers, Readers, and Erasers of DNA Modifications

Investigations into DNA modifications, particularly DNA methylation changes, are among the most common epigenetic studies conducted in asthma research [26]. Methylation is the addition of a methyl group, by DNA methyltransferase, on cytosine at position 5 with the formation of 5-methylcytosine [120]. DNMT1 and DNMT3a, as DNA methyltransferase enzymes as writer proteins, play a key role in methylating DNA, contributing to the epigenetic alterations associated with asthma pathogenesis. Methyl-CpG-binding domain protein 2 (MBD2), a “reader” protein, recognizes methylated CpG sites, while the DNA demethylating enzyme TET1 functions as an “eraser” in asthma. Their structures and functions are discussed in detail in the following section.

### 4.1. Functional Role of DNMT1 and DNMT3a in Asthma

DNMT1 is the most important enzyme which involved in the epigenetic regulation of gene expression and maintained the methylation pattern during replication. Its reduced expression can cause hypomethylation and aberrant activation of inflammatory and immune-related genes. It has been reported by Verma et al. in 2013 that overexpression of Socs3 along with low expression of DNMT1 and IL-6 in mouse models of asthma [121]. DNA methyltransferase DNMT3a regulates Th2 cell function, particularly by limiting the expression of IL-13. Loss of DNMT3a in T helper cells results in reduced DNA methylation at the IL-13 locus, along with increased levels of histone H3 lysine 27 acetylation (H3K27Ac), a marker of transcriptionally active chromatin. This epigenetic reprogramming enhances IL-13 production and contributes to exaggerated lung inflammation in allergic airway disease models. Thus, DNMT3a acts as an epigenetic brake on Th2 cytokine expression, and its deficiency may exacerbate asthma pathogenesis by promoting IL-13–driven inflammation [41].

### 4.2. Structure of DNMT1 and DNMT3a

Both DNMT1 and DNMT3s are multi-domain proteins, containing a large regulatory region in addition to the C-terminal MTase domain [122,123]. DNMT1 comprises ~1600 amino acids, with an N-terminal regulatory region covering two thirds of the sequence, a highly conserved (GK)n repeat and a C-terminal MTase domain [124]. In Ren et al. (2018) [124] paper, the regulatory region is described as containing a 300 amino acid N-terminal domain (NTD) with protein/DNA interaction sites, followed by a replication foci-targeting sequence (RFTS) domain, a CXXC zinc finger domain, and a pair of bromo-adjacent-homology (BAH) domains, as shown in their figures and text. DNMT1 RFTS domain serves as an effector module that transmits the H3K9me3 signal into DNMT1-mediated DNA methylation. The CXXC domain of DNMT1 specifically binds unmethylated CpG dinucleotides, helping localize the enzyme to target DNA regions. It interacts with both the major and minor grooves of DNA, with a loop segment (R684–Q687) making base-specific contacts. Both BAH domains are structurally associated with the MTase domain, forming an integrated structural unit [124]. The function of DNMT1 in replication-dependent DNA methylation maintenance is supported by its localization in replication foci during the S phase, and in vitro a 3–40-fold enzymatic preference for hemimethylated CpG sites [122,125], an epigenetic mark enriched at the replication foci [126]. DNMT3a and DNMT3b mediate DNA methylation establishment during gametogenesis and embryogenesis [127,128] and subsequently participate in methylation maintenance. DNMT3a and DNMT3b are highly related in sequence, both containing a largely disordered NTD, followed by a Pro-Trp-Trp-Pro (PWWP) domain, an Atrx-Dnmt3-Dnmt3l (ADD) domain and a highly homologous MTase domain.

**Figure 4:**
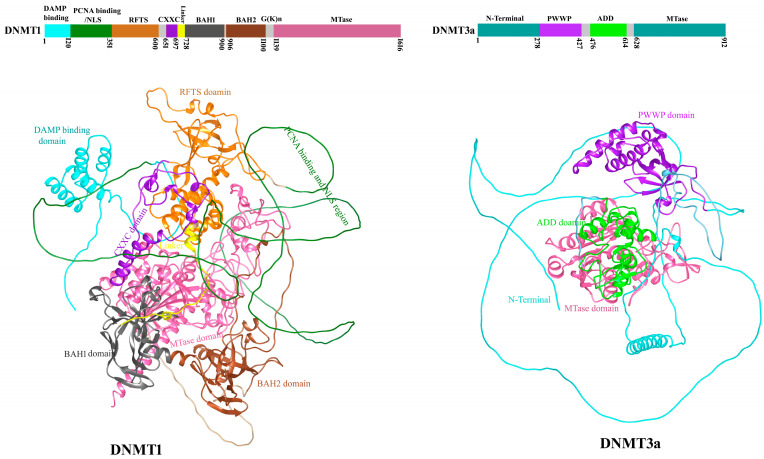
Structural domains of DNMT1 and DNMT3a. This figure shows the structural domains of DNMT1 (**left**) and DNMT3a (**right**). The domains are color-coded and labeled, with DNMT1 displaying various domains such as DAMP binding, RFTS, BAH1, BAH2, and MTase, while DNMT3a includes the N-terminal, PWWP, ADD, and MTase domains. The catalytic and regulatory domains of DNMT1 and DNMT3a work together in substrate recognition, playing a key role in epigenetic regulation linked to asthma pathogenesis. This figure was generated in this study.

The ADD domain of DNMT3a specifically recognizes unmethylated histone H3 tails at lysine 4 (H3K4me0), ensuring that DNMT3a is recruited to transcriptionally inactive regions of chromatin [129]. This recognition prevents inappropriate DNA methylation at active gene promoters. The PWWP domain in DNMT3A and DNMT3B, facilitates chromatin binding by specifically interacting with histone H3 trimethylated at lysine 36 (H3K36me3) [130]. Ren et al. (2018) [124] explored the structural basis of DNMT1 and DNMT3a-mediated DNA methylation, revealing how their catalytic and regulatory domains collaborate in substrate recognition. These insights are essential for understanding the role of DNMT1 DNMT3a in epigenetic regulation relevant to asthma pathogenesis. Since complete sequence-based experimental 3D structures of DNMT1 and DNMT3a are not available, the structures were retrieved from the AlphaFold database, with DNMT1 (ID: AF-P26358-F1) and DNMT3a (ID: AF-Q9Y6K1-F1). These models were subsequently combined and labeled based on their respective domains, as shown in Figure 4.

### 4.3. Functional Role of Methyl-CpG-Binding Domain Protein 2 in Asthma

Methyl-CpG-binding domain protein 2 (MBD2) is a key epigenetic regulator involved in the interaction between DNA methylation and chromatin remodeling. As a reader of DNA methylation, MBD2 significantly impacts processes such as stem cell differentiation and somatic cell reprogramming [131]. A recent study demonstrated that MBD2 promotes Th17 differentiation in mice through its interaction with MINK1, which regulates IL-17 secretion [132]. Further analysis highlighted MBD2′s potential role in asthma pathogenesis and allergic reactions by influencing CD4+ T cell differentiation. Additionally, MBD2 has been shown to facilitate Th17 cell differentiation by downregulating SOCS3 expression in severe asthmatic mice [133]. Notably, peripheral blood analysis in asthma patients revealed a correlation between serum MBD2 levels and Th17 cell populations, suggesting that MBD2 expression could serve as a biomarker for asthma endotypes. Furthermore, patients with severe asthma exhibited significantly higher MBD2 levels compared to those with mild to moderate disease and healthy controls, indicating a strong correlation between MBD2 expression and asthma severity [42].

### 4.4. Structure of Methyl-CpG-Binding Domain Protein 2

The MBD2 protein consists of four distinct domains, arranged from N-terminal to C-terminal: a glycine-arginine repeat region (GR), a methyl-DNA-binding domain (MBD), an intrinsically disordered region (IDR), and a coiled-coil domain (CC) [134]. MBD recognizes and binds CpG-rich DNA regions to mediate gene silencing. As the complete experimental 3D structure of MBD2 is unavailable, its structure was retrieved from the AlphaFold database (ID: AF-Q9UBB5-F1). The retrieved model, which includes all aforementioned domains, is depicted in Figure S1 with appropriate labeling. The methyl-DNA-binding domain (MBD) of MBD2 is the primary domain responsible for recognizing methylated DNA. It is a highly conserved structure within the protein family in humans and homologous species [135]. This approximately 80-amino acid domain, located between residues 145 and 225 in humans, is present in all MBD2 isoforms [136]. The MBD2_MBD_ domain has been shown to interact with methylated residues across the genome, recognizing motifs such as mCG, mCAG, mCAT, mCC, and mCT di-/tri-nucleotide sequences with varying binding affinities [137]. MBD2_MBD_ conservatively consists of four strands of β-sheet and one α-helix loop. β-sheets are centrally located and establish a finger like protrusion for specific pairing with mCpG and for the interaction with major groove of the DNA. Particularly, β2 and β3 are crucial for DNA binding, with residues like Val164, Arg166, Asp176, Tyr178, Arg188, and Ser189 contributing to DNA interaction through hydrogen bonding, electrostatic attraction, and hydrophobic contacts [135,136,138]. The intrinsically disordered region (IDR) in MBD2 has not been fully characterized, as evidenced by AF3 with ID AF-Q9UBB5-F1, which also displays disorder in this region (Figure S1). MBD2 exists in three isoforms MBD2a, MBD2b, and MBD2c(t) which differ in domain architecture and expression. MBD2a is the full-length isoform (411 aa), MBD2b lacks the N-terminal GR-rich domain (262 aa), and MBD2c(t) is C-terminally truncated (301 aa). Despite these differences, all isoforms retain the conserved methyl-DNA-binding domain responsible for DNA interaction [139].

### 4.5. Functional Role of TET1 in Asthma and Its Structural Information

DNA demethylating enzymes (TET1, TET2, and TET3), also known as “erasers”, maintain a complex dynamic equilibrium that regulates gene expression [140]. A study using a mouse model of house dust mite–induced asthma reported an altered methylome, elevated levels of 5-hydroxymethylcytosine (5-hmC), and increased TET1 expression in the lungs, suggesting a potential role for TET1 in the pathogenesis of asthma [141]. TET1 negatively regulates allergic airway inflammation by suppressing the expression of pro-inflammatory and epithelial genes such as *Il13*, *Il33*, *Il17a*, *Muc5ac*, *Egfr,* and *Tff2*, thereby limiting airway hyperresponsiveness [142]. Hypomethylation of the TET1 gene (located at 10q21.3) in the nasal epithelium has been associated with the development of asthma in children exposed to traffic-related air pollution. TET1 is responsible for converting 5-methylcytosine into several derivatives, including oxymethylcytosine, 5-formylcytosine, 5-carboxylcytosine, and 5-hydroxymethylcytosine, thereby facilitating DNA demethylation [43]. In mammals, three TET paralogs have been identified: TET1, TET2, and TET3 [143,144,145]. The full structure of TET1 includes three major functional domains: an N-terminal CXXC domain and a C-terminal catalytic domain, which consists of a cysteine-rich region and a double-stranded β-helix (DSBH) fold. The CXXC domain specifically recognizes and binds to unmethylated CpG sites, directing TET1 to gene promoters and CpG-rich regulatory elements. The cysteine-rich region stabilizes the interaction between TET1 and DNA, supporting proper positioning for catalysis. The DSBH domain, which is the enzymatically active subunit, catalyzes the oxidation of the methyl group at the 5′position of cytosine (5mC) to 5-hydroxymethylcytosine (5hmC). This dioxygenase activity is Fe^2+^- and 2-oxoglutarate (2-OG)-dependent, and is essential for DNA demethylation, a key epigenetic mechanism in gene regulation [146,147]. Notably, TET1 promotes IL-4 expression by demethylating its promoter, contributing to allergic airway inflammation, thereby linking its structural domains directly to immune dysregulation in asthma [148].

## 5. Epigenetic Regulators in Asthma: Writers, Readers, and Erasers of RNA Modifications

N6-methyladenosine (m6A) methylation is the most abundant post-transcriptional RNA modification in mRNAs, regulating gene expression and influencing RNA fate through the action of m6A “writer” (methyltransferase), “eraser” (demethylase), and “reader” (methylated reading protein) [149,150,151,152]. m6A is involved in inflammatory and lung diseases such as asthma, respiratory distress syndrome, pneumonia, and lung cancer [153,154,155,156]. Presently, m6A methylation gains more and more attention about its function and mechanism. In the asthma pathophysiological process, there is only primary probation and initiatory findings [157,158]. For example, Teng et al. in 2021 found that dysregulated or hypermethylated m6A peaks in 329 mRNAs and 150 hypomethylated m6A peaks in 143 mRNAs in asthmatic mice [159]. In addition, Dai et al. 2021 found that 5 candidate m6A regulators (FMR1, KIAA1429, WTAP, YTHDF2, ZC3HAV1) are in close contact with the risk of childhood asthma [44]. Therefore, these studies inspire us that m6A may participate in the asthma pathology.

### 5.1. Functional Role of YTHDF1 and YTHDF2 in Asthma

m6A modification mediates the recruitment of m6A readers that associate m6A-modified RNAs with mRNA processing enzymes to influence RNA export splicing, translation, and degradation [160,161,162,163]. YTHDF1 has been identified as a translation-facilitating m6A reader that recruits translation machinery to its target mRNAs in the cytoplasm [164,165]. YTHDF1 is an m6A reader protein that recognizes and binds to the m6A methylation site of *SOCS3* mRNA, consequently promoting its translation, thereby inhibiting the JAK2/STAT3 pathway, and reducing the secretion of inflammatory factors, which results in anti-inflammatory regulation [166]. This process is functionally essential for asthma, since the inhibition of IL-1β can modulate the Th17/Treg immunological imbalance and reduce neutrophil-driven airway inflammation in an ovalbumin-induced asthma model [167].

Additionally, YTHDF1′s intimate interaction with the JAK2/STAT3 signaling pathway in macrophages has been observed in septicemic rats, enabling the induction of IL-17, which disrupts the Th1/Th17 equilibrium and accelerates inflammatory reactions [168]. This pathway, associated in asthma pathology, contributes to mast cell activation, bronchial smooth muscle thickening, and airway remodeling [169,170,171]. The therapeutic potential of targeting the JAK2/STAT3 pathway has been highlighted, as inhibition of this signaling cascade can reduce neutrophil activation and improve the efficacy of corticosteroids in asthma patients [172]. Recent research demonstrates that the YTHDF1 reader protein is increased in PDGF-BB-induced airway smooth muscle (ASM) cells, promoting cell proliferation and migration, which are critical events in airway remodeling related to asthma. Cyclin D1 (CCND1), a pivotal regulator of the cell cycle, is known as a downstream target of YTHDF1 via an m6A-dependent mechanism. YTHDF1 interacts with CCND1 mRNA, enhancing its stability and expression. The m6A-dependent modulation of CCND1 by YTHDF1 provides a new mechanism in asthma etiology, suggesting possible treatment approaches aimed at the YTHDF1-CCND1 axis [173]. YTHDF1 is significantly elevated in asthmatic patients and regulates CLOCK protein translation in a m6A modification-dependent manner. Moreover, CLOCK drives NLRP3 inflammasome activation and IL-1β release, facilitating inflammatory responses in the airways [47]. This CLOCK–NLRP3 axis plays a critical role in the pathogenesis of asthma by contributing to airway inflammation and immune dysregulation. YTHDF2, a m6A reader, regulates AXIN1 mRNA stability in an m6A-dependent manner, governed through a m6A alteration introduced by WTAP. WTAP, a key component of the m6A methyltransferase complex, promotes m6A modification of AXIN1 mRNA, which reduces its stability. This methylation allows the m6A reader protein YTHDF2 to recognize and bind AXIN1 transcripts, facilitating their degradation. As AXIN1 is a negative regulator of the Wnt/β-catenin pathway, its loss results in enhanced β-catenin signaling, thereby promoting airway smooth muscle cell (ASMC) proliferation. Conversely, YTHDF2 knockdown stabilizes AXIN1 expression, suppresses β-catenin activation, and attenuates ASMC proliferation. This proposes that the YTHDF2–WTAP–AXIN1 axis plays a pivotal role in regulating ASMC growth and offers potential therapeutic targets for asthma treatment [45]. Severe asthma is a poorly controlled form of asthma, often linked to chronic rhinosinusitis with nasal polyps and frequent, drug-resistant exacerbations [174]. To explore the epigenetic basis of this condition, Sun et al. (2021) [175] conducted an integrated bioinformatics analysis using expression data from 344 severe asthma patients and 87 healthy individuals. By applying univariate logistic regression and LASSO Cox regression models, they identified YTHDF3 and YTHDC1 as key m6A regulators significantly associated with severe asthma. Both genes exhibited markedly elevated risk scores in the patient group compared to controls (*p* = 6.1 × 10^−14^), and receiver operating characteristic (ROC) curve analysis demonstrated their strong predictive performance in distinguishing severe asthma cases from healthy subjects. Furthermore, these regulators showed notable changes in their m6A methylation profiles, particularly within exonic and 3′UTR regions, suggesting their involvement in post-transcriptional RNA processing. Correlation analyses also revealed strong interactions between YTHDF3, YTHDC1, and other m6A machinery components, indicating that their cooperative activity may contribute to immune dysregulation and the pathogenesis of severe asthma. They also found that many immune cells are co-regulated by the m6A regulator, such as eosinophils, which are closely related to severe asthma, the abundance of eosinophils is positively correlated with YTHDF3 and negatively correlated with EIF3B, which proves that the expression of YTHDF3 and EIF3B has a close influence on eosinophils in the pathogenesis of severe asthma [175].

### 5.2. Structure of YTHDF Proteins

Most of these diverse functions are realized via the m6A binding proteins containing the YT521-B homology (YTH) domain, so-called m6A readers [176,177,178,179]. The experimental structural data for the DF paralogs are limited to their YTH domains [180,181]. The three human DF proteins consist of a YTH domain of 145 residues located in the sequence between unstructured N-terminal and C-terminal segments of about 400 and 35 residues, respectively [182]. The m6A-RNA-binding YTH domains of the DF paralogs span 23–24% of their length and show >90% primary sequence similarity [183]. These domains share a conserved α/β fold with six β-strands and three α-helices [180]. They fold into a β-barrel where the α-helices are packed against it to stabilize the hydrophobic and electronegative m6A-binding cage [180,181,184]. In DF1 and DF3, the adjacent C-terminus and LCD also form α-helices, which further contact the domain [180,181]. Although such folding is predicted for DF2 [185], a recent set of 19 DF2 crystals spanning its YTH domain reveal variability in the folding and configuration of this adjacent LCD region [186].

Despite their similarity, the available crystal structures reveal differences between the YTH domains of DF paralogs. The conserved α/β folds shape most of the m6A pocket; however, its outer wall forms a considerably flexible region, the m6A recognition loop. This loop contains several of the overall few non-conserved residues across these domains between DF paralogs. This loop has been suggested to easily adopt multiple conformations, to affect m6A binding affinity, and to be more flexible in DF1 via computer simulations [181]. This DF1-restricted property was attributed to the intrinsic flexibility of its non-conserved glycine 459 residue contained in the loop [181]. In addition, the surface electrostatic potential, lipophilicity, and B-factor (an estimate of thermal motion) profiles of the YTH domains demonstrate differences between paralogs [180,181]. Interestingly, the YTH domain of DF1 has a higher overall thermal stability, and this may underline differences in m6A binding affinities. Importantly, according to glide modeling of the YTH domains of the DF paralogs bound to m6A RNAs, the m6A-binding free energy appears to be lower for DF1 than for DF2 or DF3 [180,181]. This finding suggests less spontaneous binding and thus weaker m6A affinity for the DF1 YTH domain. These binding differences likely underpin the functional specialization of DF paralogs in asthma. For instance, DF3, which shows stronger m^6^A affinity, has been associated with enhanced post-transcriptional regulation of inflammation-related transcripts in severe asthma, whereas DF1′s weaker affinity may reflect a more modulatory or context-dependent role. Thus, differences in m^6^A-binding strength may translate into paralog-specific regulatory capacities in airway inflammation and asthma severity.

**Figure 5:**
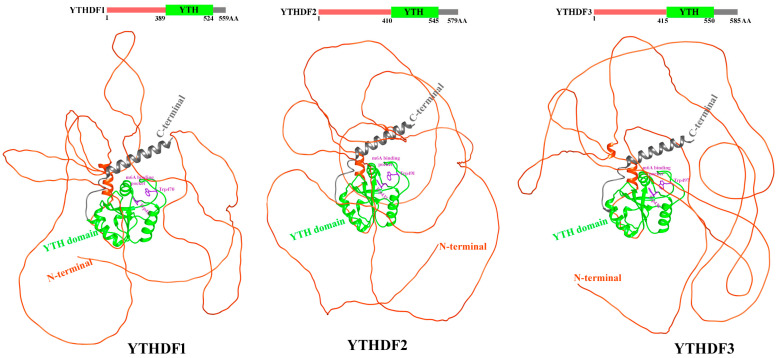
Structural comparison of YTHDF1, YTHDF2 and YTHDF3. The figure depicts the domain organization and 3D structure of YTHDF1 (**left**), YTHDF2 (**middle**) and YTHDF3 (**right**). The N-terminal is red, the YTH domain is green, and the C-terminal is dim gray. Protein lengths are indicated in amino acids (AA) at the top of each panel. Each structure highlights the conserved YTH domain (green) and the extended regions of the N-terminal (red) and C-terminal (dim gray). The m6A-binding pocket of the YTH domain (in purple) contains key residues like Trp411, Trp470 (YTHDF1), Trp432, Trp491 (YTHDF2), and Trp438, Trp497 (YTHDF3). The modified m6A mRNA binds to these regions, regulating gene expression and modulating inflammation in asthma. This figure was generated in this study.

Excluding the structurally well-characterized and highly conserved YTH domains, the DF paralog protein sequence identities demonstrate only 42–81% shared identity in their C-terminal regions and 47–64% identity in their N-terminal regions [183]. The N-terminal regions also contain sequences with <30% cross-paralog similarity that span up to 15% (85AA) of the protein [187]. YTH domain-containing proteins recognize the m6A-binding sites through a conserved aromatic cage consisting of three tryptophan residues. In this review manuscript, we analyzed the full-length AlphaFold [185], predicted structures of YTHDF1 (AF-Q9BYJ9-F1) and YTHDF2 (AF-Q9Y5A9-F1). While the N- and C-terminal regions showed low confidence scores, suggesting intrinsic disorder or flexibility, the YTH domains were modeled with high confidence, indicating well-defined and reliable structures (Figure 5). The YTH domain of DF1 consists of β1, α1, β2 from the C termini, α2 from the N-terminus, and the loop between β4 and β5 (Figure 5 (Left)). DF1 specifically recognizes m6A sites using m6A-binding pocket composed of Trp411, Trp465, and Trp470 [180]. DF2 consists of three α helices, eight β strands, and two 310 helices. The hydrophobic core consists of a β-barrel fold (β8–β1–β3–β4–β5–β2) and three α helices (Figure 5 (right)). Trp486 from the β4–β5 loop, Trp432 from the β2 strand and Trp491 from the β4–β5 loop are the basis of aromatic cage and can recognize m6A mononucleotide sites [188].

### 5.3. Functional Role of Wilms’ Tumor 1-Associating Protein in Asthma

According to Deng et al. [189], the upregulation of Wilms’ tumor 1-associating protein (WTAP) expression endorsed Wnt signaling activation and triggered the excessive growth of pancreatic ductal adenocarcinoma cells. AXIN1 referred to as an axon, is a gene cloned from a mouse mutant. AXIN1 is known as an inhibitor of the Wnt signaling pathway [190]. Abnormal activation of the Wnt/β-catenin pathway is closely related to the occurrence of asthma [191]. This mechanistic link is supported by earlier findings (see Section 5.1), where WTAP-mediated m^6^A modification of AXIN1 mRNA promotes its YTHDF2-dependent degradation, thereby enhancing Wnt/β-catenin signaling and contributing to airway smooth muscle cell proliferation in asthma. Lin et al. evaluated the consequences of RNA modification writers on the immune microenvironment in severe asthma by studying the relationship between immune cell populations and HLA gene expression alongside RNA writer modification patterns. Their findings suggest that WTAP, which was significantly increased in severe asthma, demonstrated a robust positive correlation with multiple immune cell types and HLA genes [192]. Hyperinflammation is frequently linked to elevated WTAP levels in inflammatory diseases. Numerous GEO datasets (GSE13887/137268/69063/97779/166388/208303) were investigated by Ge et al. to explore the role of m6A modification in inflammation. The findings indicated that WTAP expression is consistently elevated in patients with systemic lupus erythematosus (SLE), asthma, sepsis, rheumatoid arthritis (RA), psoriasis, and Crohn’s disease, signifying its role in the inflammatory processes of these diseases [193].

### 5.4. Structure of Wilms’ Tumor 1-Associating Protein

**Figure 6:**
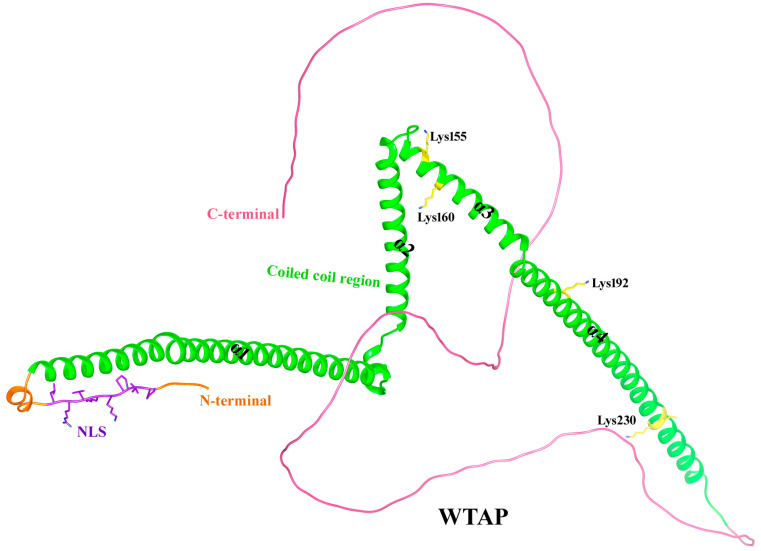
Structural representation of WTAP. The 3D structure of human WTAP is shown, highlighting its distinct functional regions. The N-terminal domain (orange) includes the nuclear localization signal (NLS) (purple), essential for WTAP’s localization to nuclear speckles. This is followed by the coiled-coil domain (green), which mediates protein–protein interactions. The C-terminal region is shown in pink. Key lysine residues (K155, K160, K192, K230) within α-helices H3 and H4 of the coiled-coil domain are labeled in yellow; these have been experimentally identified as crosslinking sites for METTL3, indicating their role in stabilizing the m^6^A writer complex. This structural organization supports WTAP’s role in facilitating selective m^6^A deposition on inflammation-related transcripts, linking its function to airway inflammation in asthma.

Wilms’ tumor 1-associating protein (WTAP), encoded at human chromosomal region 6q25.3, is a 44 kDa protein composed of 396 amino acids [194,195]. Structural studies have revealed that WTAP encompasses an elongated N-terminal, coiled-coil domain succeeded by a rather disordered C-terminal region [196]. WTAP is localized in both the nucleus and cytoplasm [197,198]. The entire structural model of the WTAP was retrieved from the AlphaFold Protein Structure Database employing the entry ID AF-Q15007-F1 (Figure 6). The WTAP forms a symmetric parallel α-helical coiled-coil homodimer through hydrophobic interactions [199]. WTAP is a key component in m6A modification, forming a complex with VIRMA, RBM15/15B, ZC3H13 (KIAA0853), CBLL1, and METTL3/14 [197]. The N-terminal region of WTAP contains a nuclear localization signal (residues 5–13; -PLPKKVRL- to -PLPGGVGL-) that directs the METTL3/14 heterodimer to nuclear speckles [196]. This region, spanning residues 1–150, also harbors the coiled-coil domain that interacts with the N-terminal leader helix (LH) of METTL3 [196]. Although WT1 was found to interact with WTAP, it was confirmed that WT1 was dispensable for the regulation of m6A modification by WTAP [198]. In the context of the METTL3-METTL14-WTAP-VIRMA complex, WTAP adopts a more compact conformation, interacting with VIRMA through three major interfaces. VIRMA, containing 17 armadillo-like repeats, forms a twisted α-solenoid structure, facilitating preferential m6A modification at 3′ UTRs. The structural integration of WTAP with the multicomponent methyltransferase complex (MTC) core enhances substrate recruitment and catalytic efficiency, underscoring WTAP’s role as a linker between VIRMA and the METTL3-METTL14 core, forming a stable quaternary complex vital for m6A methylation activity [199]. WTAP functions as a critical regulator of inflammation by promoting m6A methylation of pro-inflammatory transcripts. Upon stimulation by NF-κB p65 and inflammatory signals, WTAP expression increases and undergoes phase separation via its prion-like and structural domains. This facilitates the assembly of m6A writer complex in nuclear speckles. This enables selective m6A deposition on actively transcribed inflammatory genes such as *IL6ST*, *IL18R1*, and *IL15RA*, thereby enhancing their protein synthesis and amplifying inflammatory responses. WTAP is therefore considered a risk factor in inflammatory diseases such as sepsis and asthma and represents a potential therapeutic target for reducing excessive inflammation [193].

### 5.5. Functional Role of METTL3 and METTL14 in Asthma

The overexpression of METTL3 in asthma models helps to increase the m6A modification of GPX4, a key protein that protects cells from ferroptosis (a type of cell death). By upregulating GPX4, METTL3 inhibits ferroptosis in bronchial epithelial cells, which promotes cell survival, proliferation, and alleviates asthma symptoms. Therefore, METTL3 overexpression plays a protective role in asthma by preventing ferroptosis and supporting cell function [200]. Previous studies have shown that METTL3 reduces and negatively regulates Th2 cell differentiation in allergic asthma. In T2 asthma, a marked decrease in METTL3 expression was observed at clinical, cellular, and animal levels, correlating with increased disease severity and airway inflammation. Low METTL3 levels promote inflammation and exacerbate asthma by activating M2-type macrophages. Additionally, silencing METTL3 in antigen-presenting HBE cells enhanced Th2 cell differentiation, underscoring METTL3′s critical role in regulating the immune response and airway inflammation in T2 asthma. METTL3 is the catalytically active component of the m^6^A writer complex, METTL14 primarily contributes to RNA substrate recognition and complex stability, as supported by structural studies [201]. Although direct evidence for METTL14′s role in ferroptosis and Th2 immunity in asthma is limited, its scaffolding function is essential for guiding METTL3-mediated m^6^A deposition on inflammation- and epithelium-related transcripts. These findings highlight METTL3 as a key modulator of asthma pathogenesis [202]. Remarkably, recent research reported that the m6A writer METTL14 is decreased significantly both in mRNA and protein level in lung of mice with allergic asthma compared with control [203]. In addition to its structural role within the m^6^A writer complex, recent studies suggest that METTL14 modulates the immune microenvironment in respiratory allergic diseases. Although catalytically inactive, METTL14 influences m^6^A deposition specificity by stabilizing METTL3 and guiding RNA substrate recognition. Bioinformatics analysis indicates METTL14 is associated with pathways such as glutathione derivative metabolism, calcium signaling, and autophagy regulation, all of which are implicated in inflammation and epithelial stress responses. Moreover, METTL14 expression correlates with patterns of immune cell infiltration, highlighting its potential impact on airway immune homeostasis. These findings point to METTL14 as a putative therapeutic target, potentially responsive to nasal methylprednisolone treatment in allergic airway conditions [204].

### 5.6. Structure of METTL3 and METTL14

**Figure 7:**
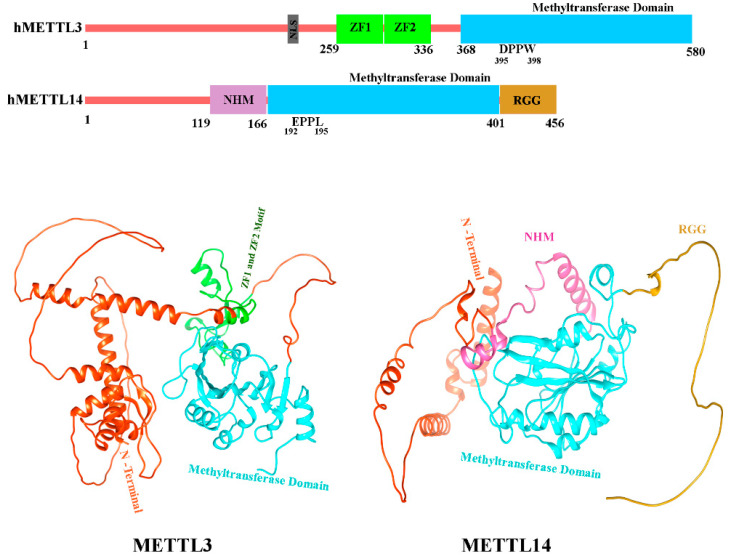
Domain architecture and 3-dimensional illustration of METTL3 and METTL14. The panel on the top depicts the domain structure of human METTL3 and METTL14. The key domains are marked. The lower panel illustrates the 3D structures of METTL3 (**left**) and METTL14 (**right**), with the N-terminal, Methyltransferase Domain, and other significant domains accentuated in different colors for clarity. The protein structures are visualized to demonstrate the spatial arrangement of the domains.

In humans, two methyltransferases, METTL3 (sometimes stated as MT-A70) and METTL14, function as core writers in m6A modification [205,206]. Both enzymes belong to the class I MTase family [207], and they form a core catalytic complex that is regulated by an additional subunit, Wilms’ tumor 1-associating protein (WTAP) [208]. Independently, METTL3 and METTL14 demonstrate minimal in vitro methyltransferase activity and need to be in complex with one another for optimal catalytic activity. Interestingly, catalytic activity requires full-length METTL3, while only the central methyltransferase domain of METTL14 is required [209]. Structurally, METTL3 comprises an N-terminal low-complexity region with a nuclear localization signal (NLS), followed by a zinc finger domain with two consecutive CCCH motifs (ZF1 and ZF2) that mediate RNA substrate binding. This region is connected via a flexible linker to the C-terminal methyltransferase domain, which adopts a classical Rossmann fold architecture (Figure 7) [201,210]. METTL14, by contrast, contains a non-functional methyltransferase domain which is bordered by an N-terminal helical motif (NHM) and a C-terminal arginine-rich region (RGG; Figure 7) [211]. METTL3′s Rossmann fold holds a catalytic loop with the classical DPPW motif [212], while METTL14 has a nonfunctional EPPL motif (Figure 7). The ZF1/ZF2 motifs of METTL3 recognize and position the DRACH consensus sequence, while RNA binding requires cooperative action of both subunits [209]. The RGG domain of METTL14 contributes additional positive charges that enhance RNA interaction [196], and together the METTL3–METTL14 dimer forms a positively charged groove at their protein–protein interface, a structural feature critical for stabilizing mRNA binding and achieving full methyltransferase activity [201]. These structural motifs mainly METTL3′s Rossmann fold and zinc finger (ZF) domains are functionally relevant to asthma, as they enable specific m^6^A deposition on epithelial transcripts such as *GPX4* [200]. These m^6^A marks enhance GPX4 expression, which plays a protective role by regulating ferroptosis resistance and maintaining epithelial cell survival key processes in mitigating airway inflammation. Disruption in these structural domains or methylation patterns has been linked to increased airway epithelial damage and heightened asthma severity. More in-depth analysis of the structural aspects of METTL3/14 RNA binding can be found in [209,213,214].

### 5.7. Role of IGF2BP2 in Asthma

IGF2BPs have recently been noted for their role in modulating biological processes including as development, cancer, and stemness. Recent discoveries reveal that IGF2BP2 is crucial for the recognition of m6A alterations and significantly affects mRNA stability and translation [215]. The significance of IGF2BP2 in macrophage polarization as a m6A reader is still ambiguous. Wang et al. (2021) [49] observed that IGF2BP2 expression raised through macrophage differentiation into M1 and M2 phenotypes, with IL-4-induced overexpression being dependent on STAT6. Their loss-of-function investigations suggested that IGF2BP2 favorably regulates IL-4-driven M2 activation while negatively impacting pro-inflammatory responses. Furthermore, m6A-RIP-qPCR identified TSC1 as a m6A-modified target recognized by IGF2BP2, pointing to its role in macrophage polarization [49]. This indicates that IGF2BP2 facilitates M2 macrophage polarization, hence intensifying allergic inflammation in asthma. M2 macrophages contribute to eosinophilic inflammation by secreting chemokines such as CCL17 and CCL22, which recruit eosinophils into the airway, thereby exacerbating allergic asthma symptoms.

### 5.8. Structure of IGF2BP2

The m6A reader protein IGF2BP2 identifies and binds to RNA via its unique structural configuration, which consists of six specific RNA-binding domains. This structure has two RNA recognition motifs (RRM1 and RRM2) and four K Homology (KH) domains (KH1–KH4) [216]. Structural insights into IGF2BP2 were derived from the protein structure predicted by AlphaFold3, accessible in the AlphaFold Protein Structure Database (ID: AF-F8W930-F1). The KH domains within IGF2BPs, specifically the KH3-4 domains, have been verified as the critical elements responsible for recognizing and binding to m6A-modified RNA [215]. These KH domains identify short RNA motifs of 3–6 nucleotides through their conserved GXXG motifs, which are positioned within a characteristic flexible loop [217]. In IGF2BP2, the KH4 domain contains a conserved 503GKGG506 motif, along with adjacent hydrophobic residues that together form a persistent hydrophobic groove. This structural configuration is essential for the selective recognition of the m6A-modified RNA, as demonstrated by Fakhar et al. (2024) [218]. This structural basis underpins IGF2BP2′s ability to recognize m6A-modified transcripts such as TSC1, allowing it to stabilize TSC1 mRNA and modulate macrophage polarization, thus linking RNA structural specificity to immune function in allergic asthma [49].

### 5.9. Role of FTO in Asthma

Lian et al. investigated that Fat mass and obesity—associated protein (FTO), as a m6A demethylase, is pivotal in asthma through modulating inflammatory responses in epithelial cells. Using the small-molecule inhibitor FB23, they demonstrated that inhibiting FTO alleviates allergic inflammation in both in vitro epithelial cell models and an in vivo house dust mite-induced asthma mouse mode [219]. FTO plays a critical role in modulating asthma exacerbation induced by PM 2.5 exposure. FTO expression is upregulated in asthmatic mice and cells exposed to PM2.5, primarily through reactive oxygen species (ROS)-dependent mechanisms, which contribute to airway epithelial barrier damage. The regulation of inhibitor of nuclear factor kappa B kinase subunit beta (IKBKB) by FTO, via m6A-dependent mRNA stability, further exacerbates inflammation through NF-κB signaling. These findings highlight the potential therapeutic benefit of targeting FTO or utilizing antioxidants to mitigate PM2.5-induced asthma exacerbation [50]. Studies in mouse models revealed that FTO knockdown accelerates airway inflammation by disrupting the balance of key cytokines and impairing epithelial function. Specifically, IL-13, a central mediator of type 2 (Th2) immune responses, is significantly upregulated. IL-13 promotes airway hyperresponsiveness by inducing the contraction of airway smooth muscle cells and stimulating mucus secretion from epithelial cells hallmark features of allergic asthma [220]. In contrast, IL-12b expression is downregulated. IL-12b encodes the p40 subunit shared by IL-12 and IL-23, which are essential for promoting Th1 and Th17 immune responses, respectively. These pathways normally function to counteract excessive Th2-driven inflammation, and their suppression contributes to immune imbalance. This cytokine dysregulation is likely linked to FTO’s regulation of m^6^A methylation on Foxj1 mRNA, a master transcription factor responsible for motile cilia formation in airway epithelial cells. Loss or reduction in Foxj1 expression impairs cilia development and coordination, resulting in defective mucociliary clearance, a key mechanism for maintaining airway hygiene and epithelial integrity. Collectively, altered cytokine signaling combined with impaired ciliary function due to Foxj1 downregulation contributes to epithelial dysfunction and chronic airway inflammation in asthma [154].

### 5.10. Structure of FTO

**Figure 8:**
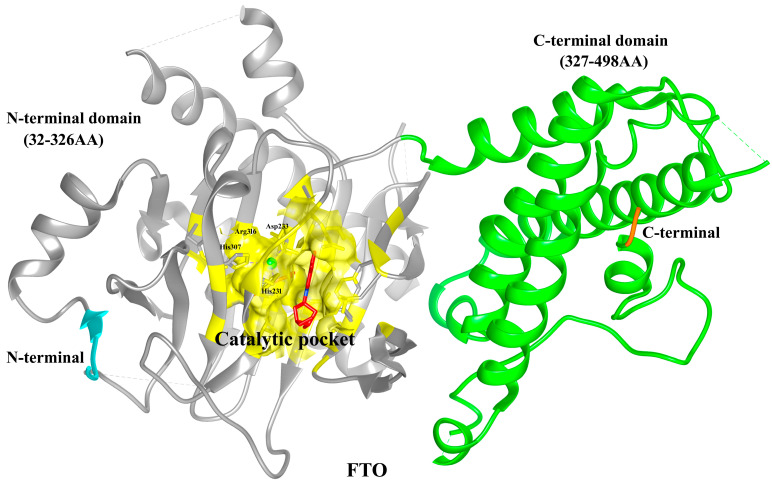
Structural domains of the human FTO protein. Ribbon representation of the full-length human FTO protein structure, highlighting its two major domains. N and C terminal labeled accordingly. The N-terminal domain (residues 32–326, shown in gray) contains the catalytic pocket, with key residues that coordinate Fe(II) and 2-oxoglutarate, both of which are essential for m^6^A demethylation. In contrast, the C-terminal domain (residues 327–498, shown in green) contributes to structural stability. The N- and C-termini are labeled accordingly. In the context of asthma, structural characterization of these domains is essential, as FTO’s demethylase activity regulates cytokine expression.

The fat mass and obesity associated gene (FTO) encodes 505-amino-acid-long protein which belongs to the AlkB family of non-heme iron and 2-oxoglutarate (2-OG)-dependent dioxygenases. This family of enzymes assists in demethylation activities, indicating the valuable function of FTO in oxidative reactions that demethylate RNA [221]. A large number of the human FTO protein X-ray crystallographic structures have been added to the Protein Data Bank (PDB) (https://www.rcsb.org, accessed on 5 January 2025) revealing thorough structural information about the protein’s catalytic processes, substrate selectivity, and interactions against various ligands and inhibitors [222,223,224,225,226,227]. Two separate domains can be observed in the crystal structure of FTOΔ31 (PDB ID:3LFM): the C-terminal domain (CTD; 327–498 AA) and the N-terminal domain (NTD; 32–326 AA). Understanding the structure of FTO’s catalytic pocket and enzymatic activity has been made possible by its crystal structure [224]. We used the crystal structure of FTO (PDB ID: 3LFM) as a reference to annotate all structural domains and highlight the catalytic pocket within the N-terminal domain of protein (Figure 8). The N-terminal domain (NTD), which contains the catalytic pocket formed by conserved residues (His231, Asp233, His307, and Arg316) that coordinate Fe(II) and 2-oxoglutarate, essential for m^6^A demethylation. The C-terminal domain (CTD) stabilizes the overall fold and may contribute to substrate orientation. This domain-specific understanding of FTO may help inform therapeutic strategies aimed at either inhibiting or enhancing its enzymatic activity depending on disease context.

## 6. Conclusions and Perspective

This study highlights the central role of epigenetically important regulators classified as writers, erasers, and readers of histone, DNA, and RNA modifications in asthma pathogenesis. Dysregulation of histone acetyltransferases (e.g., p300/CBP) and deacetylases (e.g., SIRT1–SIRT7) alters chromatin accessibility, leading to pro-inflammatory gene expression. DNA methylation enzymes such as DNMT1 and DNMT3A, along with demethylases like TET1 and readers like MBD2, modulate immune gene profiles. Additionally, m6A RNA modifiers METTL3/14 (writers), FTO (eraser), YTHDF and IGF2BP2 proteins (readers) emerge as pivotal regulators affecting asthma susceptibility and severity by influencing inflammatory pathways, immune response modulation, and airway remodeling. Structurally, these proteins feature unique and conserved domains crucial for their catalytic and regulatory functions, as highlighted by detailed protein domain analyses, sequence similarities, and structural predictions presented in the manuscript. These domain-specific structural insights not only deepen our mechanistic understanding of epigenetic dysregulation in asthma but also provide a rational basis for drug targeting. The asthma-specific impact of these regulators further emphasizes the need for precision diagnostics and stratified therapeutic strategies. Understanding these proteins’ molecular mechanisms and their interactions could uncover novel biomarkers for asthma diagnosis and management and open promising avenues for targeted therapies. Therapeutically, targeting these epigenetic regulators offers promising strategies to reverse aberrant gene expression and improve asthma outcomes. HDAC modulators, DNA methylation inhibitors, and m6A-targeted drugs may complement or enhance existing therapies. Future research should focus on phenotype-specific epigenetic profiling, structure-based drug design, and combinatorial approaches that integrate epigenetic and immunological interventions to achieve precision medicine in asthma care.

## Figures and Tables

**Table 1:** Epigenetic regulators in asthma: writers, readers and erasers of Histone, DNA and RNA modifications.

Modification Type	Role	Examples	Function	References
Histone Modification	Writer	p300/CBP	p300 and CBP are histone acetyltransferases that, with increased expression in asthma, likely activate pro-inflammatory genes, contributing to chronic airway inflammation	[29]
		KAT2A	KAT2A plays a crucial role in acetylating lysine 18 on histone 3, a modification that is found to be elevated in the epithelial cells of individuals with asthma	[30]
		SMYD3	SMYD3 was found to be upregulated at the mRNA level in airway fibroblasts from asthmatic individuals, suggesting its involvement in asthma-related epigenetic dysregulation	[31]
	Eraser	HDAC1	HDAC1 was significantly increased in bronchial epithelial cells (HBECs) of asthmatic patients	[32]
		HDAC2	Patients with mild asthma exhibit a slight decrease in HDAC2 activity in bronchial biopsies and alveolar macrophages	[33]
		HDAC3	HDAC3 regulates NF-κB activity in asthma by deacetylating specific lysine residues, suppressing inflammation. HDAC3 deficiency in macrophages reduces inflammatory gene expression, underscoring its role in controlling asthma-related inflammation	[34]
		SIRT1	Both protective and deleterious roles in asthma	[35]
		SIRT2	SIRT2 exacerbates asthma-associated inflammation by driving Th2 cell responses and macrophage polarization	[36]
		SIRT3	Song et al. found that decreased Sirt3 expression in asthmatic mice contributes to increased apoptosis, oxidative stress, and inflammation	[37]
		SIRT6	Jang et al. found that Sirt6 is upregulated in asthmatic mice	[38]
		SIRT7	Fang et al. found that increased SIRT7 expression in airway smooth muscle cells regulates TGF-β1-induced cell proliferation and migration, highlighting its role in asthmatic airway remodeling	[39]
DNA modification	Writer	DNMT1	DNMT1 maintains DNA methylation patterns, and reduced levels are associated with increased Socs3 expression, promoting inflammation in asthma	[40]
		DNMT3a	Dnmt3a regulates Th2 responses by modulating IL-13 gene methylation; loss of Dnmt3a decreases methylation, enhancing IL-13 expression and asthma-associated lung inflammation	[41]
	Reader	MBD2	MBD2 is an epigenetic reader protein recognizing methylated CpG sites, suppressing SOCS3 expression, and promoting Th17 cell differentiation. Elevated MBD2 drives neutrophilic inflammation, contributing to severe asthma	[42]
	Eraser	TET1	Reduced TET1 promoter methylation (cg23602092) in nasal cells correlates with childhood asthma and traffic-related air pollution, altering TET1 expression and 5hmC. TET1 modulates DNA methylation and epigenetic regulation in asthma	[43]
RNA modification	Writer	WTAP	WTAP was demonstrated to be abnormally expressed in asthma patients WTAP knockdown relieves asthma progression by regulating the m6A levels of AXIN1 in a YTHDF2-dependent manner	[44,45]
		METTL3	METTL3 regulates Th2 cell differentiation in T2 asthma by modulating SOX5 m6A methylation in bronchial epithelial cells. This mechanism may offer a potential target for preventing and managing T2 asthma	[46]
	Reader	YTHDF1	YTHDF1, highly expressed in airway epithelial cells of allergic and asthmatic individuals, enhances CLOCK translation in an m6A-dependent manner. This triggers NLRP3 inflammasome activation and IL-1β secretion, promoting inflammatory responses in the airways	[47]
		YTHDF2	m6A-YTHDF2 regulates macrophage polarization by inhibiting M1 and promoting M2 phenotypes through NF-κB, MAPK, and STAT pathways, playing a key role in asthma subtypes and targeted therapy	[48]
		IGF2BP2	IGF2BP2 promotes asthma by stabilizing Tsc1 mRNA, which helps macrophages adopt the M2 phenotype	[49]
	Eraser	FTO	FTO plays a pivotal role as an eraser of m6A modifications in asthma by regulating the stability of mRNA transcripts such as IKBKB, leading to the activation of the NF-κB pathway and contributing to inflammation and epithelial barrier dysfunction	[50]

## Data Availability

Not applicable.

## Supplementary Materials

The following supporting information can be downloaded at: https://www.mdpi.com/article/10.3390/biom15091255/s1, Figure S1: 3D structure of MBD2 protein with domain annotation. AlphaFold 3-based predicted structure of MBD2 showing its distinct functional domains, each color-coded and labeled accordingly.

## Abbreviations

The following abbreviations are used in this manuscript:

CBP	CREB binding protein
SIRT	Silent information regulator proteins
DNMT1	DNA (cytosine-5)-methyltransferase 1
IGF2BP2	Insulin-like growth factor 2 mrna-binding protein 2
MBD2	Methyl-CpG-binding domain protein 2
TET1	Ten-eleven translocation methylcytosine dioxygenase 1
FTO	Fat mass and obesity associated gene
RNA	Ribonucleic acid
DNA	Deoxyribonucleic acid
PTMs	Posttranslational modifications
HATs	Histone acetylases
HDACs	Histone deacetylases
TFs	Transcription factors
METTL3	Methyltransferase-like 3
WTAP	Wilms’ tumor 1-associating protein
IBiD	interferon-binding transactivation domain
PWWP	Pro-Trp-Trp-Pro
ADD	Atrx-dnmt3-dnmt3l
DSBH	double-stranded β helix
IL-17	Interleukin-17
ASMC	Airway smooth muscle cell
ZF1	Zinc finger motifs
RRM1	RNA recognition motif 1
RRM2	RNA recognition motif 2
PTM	Posttranslational modifications
EWAS	Epigenome-wide association studies
miRNAs	microRNAs
lncRNAs	Long non-coding RNAs
m6A	N6-methyladenosine
KAT2A	Lysine acetyltransferase 2A
SMYD3	SET and MYND domain-containing protein 3
SIRT1/2/3/6/7	Sirtuin 1/2/3/6/7
ORMDL3	Orosomucoid-like protein 3
EMT	Epithelial–mesenchymal transition
ARDS	Acute respiratory distress syndrome
NRID	Nuclear receptor interaction domain
NF-Kb patway	Nuclear factor kappa-light-chain-enhancer of activated B cells pathway
NOD	Nucleotide-binding oligomerization domain
NAD+	Nicotinamide adenine dinucleotide
H3K27Ac	Histone H3 lysine 27 acetylation
CpG	Cytosine–phosphate–guanine
BAH	Bromo-adjacent-homology
STAT3	Signal transducer and activator of transcription 3
JAK2	Janus kinase 2
CCND1	Cyclin D1
NLRP3	NOD like receptor thermal protein domain associated protein 3
AXIN1	axis inhibition protein 1
ROC	Receiver operating characteristic
YTHDF1/2/3	YTH domain-containing family protein 1/2/3
METTL14	Methyltransferase like 14
CLOCK	Circadian locomotor output cycles kaput
5mC	5-methylcytosine
5hmC	5-hydroxymethylcytosine
Th1/Th2/Th17	T helper type 1/2/3
3′UTR	3′ Untranslated region
EIF3B	Eukaryotic translation initiation factor 3 subunit B
LCD	Low complexity domain.
GEO	Gene expression omnibus
SLE	Lupus erythematosus
RA	Rheumatoid arthritis
NLS	Nuclear localization signal
MTC	Methyltransferase complex
RGG	Arginine–Glycine–Glycine
m6A-RIP-Qpcr	N6-Methyladenosine RNA Immunoprecipitation followed by quantitative Polymerase Chain Reaction
CCL17/22	The C-C class chemokines 17/22
IKBKB	Inhibitor of nuclear factor kappa B kinase subunit beta
CTD	C-terminal domain
NTD	N-terminal domain
