# Supplementary figures and images for: Structural and Functional Studies on Key Epigenetic Regulators in Asthma
Source: Biomolecules. 2025 Aug 29;15(9):1255. doi: 10.3390/biom15091255 (PMC12466977; doi:10.3390/biom15091255)

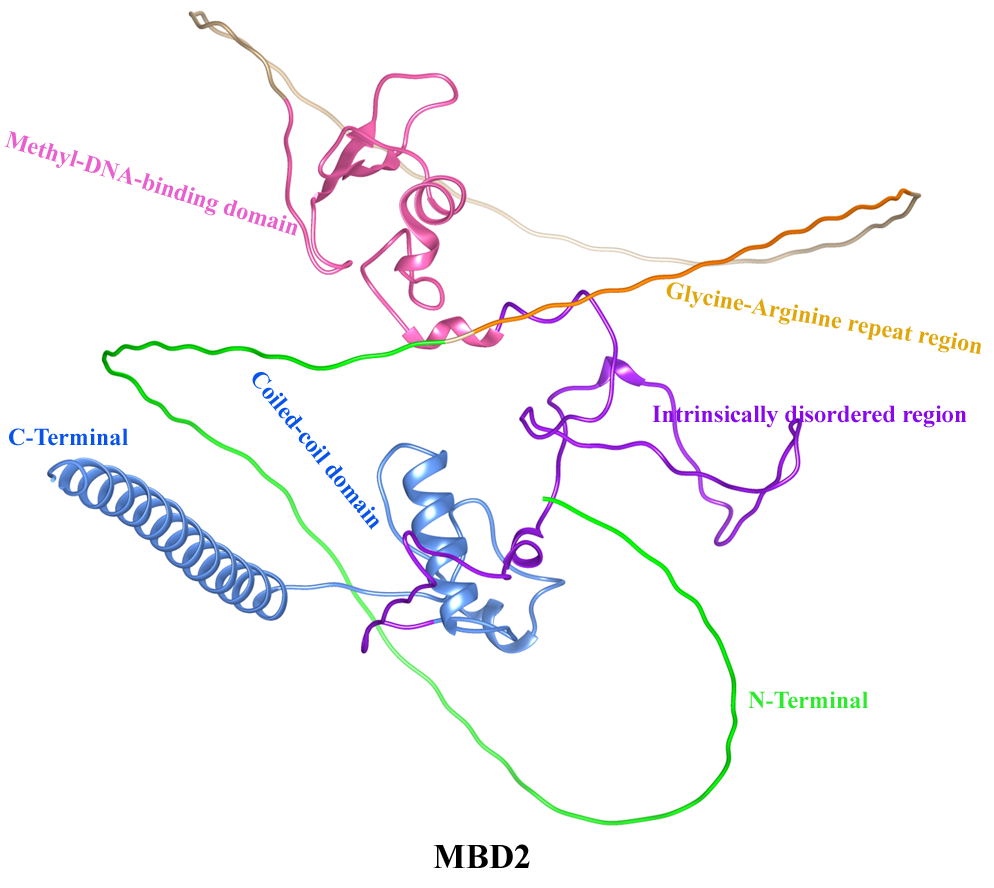

Supplement: Supplementary file 1 [file biomolecules-15-01255-s001.zip › biomolecules-3650474-Figure S1.tif]
